# Coinfection patterns and associations of tick-borne pathogens in natural foci of severe fever with thrombocytopenia syndrome: A One Health approach

**DOI:** 10.1016/j.onehlt.2026.101543

**Published:** 2026-09-17

**Authors:** Zhiyi Zhang, Bingzhi Chen, Yishuang Zhao, Xiang Zheng, Qing Gao, Hongbiao Liang, Haijiang Lin, Na He

**Affiliations:** a Shanghai Institute of Infectious Disease and Biosecurity, Fudan University, Shanghai, China; b School of Public Health, Fudan University, Shanghai, China; c Key Laboratory of Public Health Safety of Ministry of Education, Fudan University, Shanghai, China; d Taizhou City Center for Disease Control and Prevention, Zhejiang Province, China

**Keywords:** Tick-borne pathogens, Coinfection, Association, One Health

## Abstract

**Background:**

Tick-borne pathogens (TBPs) circulate in complex ecological systems involving vectors, animal hosts, and humans. Coinfections have been shown to influence clinical outcomes and may complicate pathogen associations, yet current research remains limited.

**Methods:**

We adopted a One Health approach in Taizhou City, a severe fever with thrombocytopenia syndrome (SFTS) endemic area, by collecting 485 community residents, 90 confirmed SFTS patients, 1081 ticks, and 471 small mammals. Five TBPs, including *Anaplasma* spp., *Bartonella* spp., *Coxiella* spp., *Ehrlichia* spp., and spotted fever group rickettsiae (SFGR), were detected using indirect immunofluorescence assay or polymerase chain reaction. We subsequently analyzed pathogen prevalence and coinfection patterns, assessed pathogen-pathogen associations, and evaluated cross-host correlations at the township level.

**Results:**

All five TBPs were detected across ticks, small mammals, and humans, with prevalence varying substantially. Human IgG showed diverse co-seropositivity, while coinfections in ticks and small mammals were limited. Significant pathogen-pathogen associations were observed: *Anaplasma* spp./*Ehrlichia* spp. and *Ehrlichia* spp./SFGR in humans, and *Anaplasma* spp./*Coxiella* spp. in small mammals. Cross-host correlations included positive associations of SFGR in ticks with community residents, *Bartonella* spp. in small mammals with community residents, and significant links of *Anaplasma* spp./*Ehrlichia* spp. and *Anaplasma* spp./SFGR between ticks and community residents.

**Conclusions:**

Multiple TBPs co-circulate in this SFTS epidemic area, following distinct exposure routes involving ticks and small mammals. *Anaplasma* spp., *Ehrlichia* spp., and SFGR are tick-borne, while *Bartonella* spp. is more associated with small mammals. IgG co-seropositivity in humans likely reflects repeated or long-term exposures rather than a single tick bite. These findings suggest that effective prevention should consider human activity patterns and the role of small mammals in sustaining pathogen circulation, while a One Health approach integrating humans, animals, and vectors can guide the development of more targeted control strategies.

## 1 Introduction

Ticks are among the earliest arthropods identified as vectors of pathogens [1]. They can acquire, maintain, and transmit a wide range of pathogens and are distributed across most regions of the world. The geographic diversity of tick species contributes substantially to regional variation in the prevalence of tick-borne pathogens (TBPs) [2]. For instance, severe fever with thrombocytopenia syndrome virus (SFTSV) has been reported primarily in East Asia, particularly in China and South Korea [3], [4]. By contrast, spotted fever group rickettsiae (SFGR) are globally prevalent but exhibit considerable species-level heterogeneity [5]. For example, *Rickettsia rickettsii* causes Rocky Mountain spotted fever in the United States [6], whereas *Rickettsia conorii* subsp. *conorii* is is associated with Mediterranean Spotted Fever in the Mediterranean region [7], [8]. In China, multiple SFGR have been reported to infect humans, including common species such as *Rickettsia raoultii*, *Rickettsia monacensis*, and *Rickettsia japonica*, as well as emerging or newly identified SFGR such as *Rickettsia heilongjiangensis* and *Rickettsia* sp. XY99 [5], [9], [10]. The transmission of TBPs involves complex cycles that include both ticks and vertebrate hosts, which interact within a constantly changing environment. Disrupting these transmission cycles is challenging, thereby complicating the control and prevention of tick-borne diseases [1].

Beyond their complex transmission cycles, tick-borne diseases are further complicated by coinfections. Coinfection is defined as the simultaneous presence of two or more pathogens, acquired through a single tick bite or multiple sequential exposures from one or more ticks [11]. These pathogens may interact either synergistically or antagonistically, potentially modifying disease severity, complicating diagnosis, and influencing treatment strategies [12], [13]. For instance, patients with Lyme disease who are coinfected with *Babesia* spp. or *Anaplasma phagocytophilum* frequently present with more severe clinical manifestations and prolonged disease duration [11], [14], [15]. As early as in the 1970s, researchers in Norway documented coinfection of *Tick-borne encephalitis virus* (TBEV) and *Uukuniemi virus* (UUKV) in *Ixodes ricinus* ticks [16]. More recently, a study in the United States involving 16,080 ticks identified *Borrelia burgdorferi* sensu stricto and *A. phagocytophilum* as the most frequent coinfection pattern [17].

Although these studies demonstrate TBP coinfections, they have primarily focused on pathogens most relevant to human health, particularly *B. burgdorferi* sensu lato, *Babesia* spp., and *A. phagocytophilum* [18], [19], [20], [21], and are limited in geographic scope and generalizability. Furthermore, these studies have largely focused on isolated components of the tick–animal–human transmission cycle, which has constrained our understanding of pathogen associations and transmission dynamics among TBPs. Adopting a One Health approach is therefore essential, as it enables the integration of data from humans, animals, and vectors within their shared ecosystems [22]. This concept, increasingly recognized by international health agencies, is particularly relevant for TBPs, which persist within complex ecological cycles involving vectors and multiple vertebrate hosts [1], [23], [24]. By integrating tick biology, host interactions, and environmental factors, the One Health approach not only enhances scientific understanding but also strengthens public health preparedness by informing more effective surveillance, prevention, and control strategies [25], [26].

In this study, we adopted a One Health approach by simultaneously collecting tick, animal, and human samples from an epidemic area of severe fever with thrombocytopenia syndrome (SFTS) and detected five most concerned TBPs using serological and molecular methods. This approach enabled us to characterize coinfection patterns across ticks and different hosts, evaluate pathogen–pathogen associations, and explore the ecological concordance of coinfections. These findings provide new insights into the coinfection patterns and associations of TBPs across different hosts.

## 2 Methods

### 2.1 Study design

This study, conducted in Taizhou City which is an epidemic area of SFTS in Zhejiang Province, Southeastern China, was designed as a comprehensive cross-sectional survey among all confirmed SFTS inpatients, purposively selected community residents, concurrently collected ticks and trapped small mammals in the communities during the study period. Infections with five most concerned TBPs including *Anaplasma* spp., *Bartonella* spp., *Coxiella* spp., *Ehrlichia* spp., and SFGR were determined by seropositivity of IgM and IgG antibodies using indirect immunofluorescence assays (IFA) for human subjects or by detectable pathogen DNA using polymerase chain reaction (PCR) for ticks and small mammals. This study was approved by the Institutional Review Board of Taizhou Center for Disease Control and Prevention (Approval No. IRB#2020041501). Written informed consent for participation and blood sample collection was obtained from all participants. Ticks and animal sampling was conducted as part of the approved routine surveillance program in Taizhou City.

### 2.2 Enrollment and sample collection of community residents

Community residents were recruited from six townships of Taizhou City from July 2020 to June 2021, resulting in a total of 1117 participants. The detailed methods were described in a previous publication [27]. Each participant completed a structured questionnaire and provided a serum sample at enrollment. To address the research question, we first identified all individuals who self-reported at least one tick bite in the past year as the tick-bite group. Subsequently, individuals without reported tick exposure were assigned to the no tick-bite group through age- and sex-matching. Thus, the final sample for the serological study was 485 community residents.

### 2.3 Definition and sample collection of SFTS patients

According to the 2010 edition of China's national guideline for SFTS, a laboratory-confirmed diagnosis requires meeting at least one of the following criteria: (1) isolation of SFTSV from clinical specimens; (2) detection of SFTSV RNA in serum or whole blood; or (3) a four-fold or greater increase in virus-specific IgG and/or IgM antibody titers between acute- and convalescent-phase serum [28]. For this study, we included all newly reported laboratory-confirmed SFTS patients from 1 January 2020 to 31 December 2023. Case surveillance was carried out by local Center for Disease Control and Prevention and medical institutions. Following laboratory confirmation, demographic information and biological specimens-including acute-phase serum and whole blood-were collected from each patient. All specimens were transported under cold-chain conditions and stored at −80 °C until further analysis.

### 2.4 Tick collection and small mammal capture

Tick samples were collected between March and October each year from 2021 to 2024. Questing ticks were obtained using the standard flagging method, in which white woolen flannel cloths (1.2 m × 1.0 m) [27] were swept over vegetation, whereas feeding ticks were directly collected from domestic animals. All collected ticks were morphologically identified to determine species and life stages, and subsequently stored at −80 °C until further processing.

Small mammals were captured from 2021 to 2024 by qualified pest control companies commissioned by the Taizhou Center for Disease Control and Prevention, using baited cages equipped with treadle release mechanisms [27]. Traps were placed in both wild habitats and residential areas before sunset and retrieved in the following morning. Captured animals were anesthetized prior to morphological examination. Species and sex were determined based on external morphological characteristics according to the *Handbook for Identification of Important Medical Animals in China* [29]. Animals were subsequently euthanized by cervical dislocation in accordance with the *Laboratory Animal-Guidelines for Euthanasia* (https://openstd.samr.gov.cn/bzgk/std/newGbInfo?hcno=07720991C7CF4242103453E8BC3F6684). Under sterile conditions, spleen and liver tissues were collected from captured animals and preserved at −80 °C until further processing.

### 2.5 Serological detection of tick-borne pathogens for humans

In community residents, IgG antibodies against five TBPs were detected using IFA IgG antibody kits (Fuller Laboratories, Fullerton, CA, USA) following the manufacturer's instructions. In SFTS patients, both IgM and IgG antibodies against the same five TBPs were detected using the corresponding IFA kits from the same manufacturer (Fuller Laboratories, Fullerton, CA, USA). Serum samples were diluted to 1:64 for all pathogens except for *Coxiella* spp., which was tested at a 1:16 dilution. Fluorescence signals were evaluated with an inverted fluorescence microscope (DMI3000 B, Leica Microsystems, Wetzlar, Germany) by comparison with positive controls, consisting of the titration endpoint and two verification dilutions (±1 dilution from the endpoint). All slides were independently read and interpreted by two trained investigators to ensure consistency and reduce subjectivity. Detailed kit specifications and interpretation criteria are summarized in Supplementary Table 1.

### 2.6 Molecular detection of tick-borne pathogens for ticks and mammals

For each sample, either a combined total of less than 30 mg of spleen and liver tissue from small mammals or a single tick was placed into a collection tube. Following the addition of lysis buffer, samples were homogenized using a TissueLyser Mixer Mill MM 400 (Retsch GmbH, Haan, Germany). Total DNA was extracted from the homogenate using a commercial DNA Isolation Kit (TIANGEN Biotech Co., Ltd., Beijing, China), following the manufacturer's protocol. DNA was eluted in a final volume of 40 μL elution buffer and stored at −80 °C until use.

*Bartonella* spp. was detected by conventional PCR, while nested PCR was employed for all other pathogens. All amplification reactions were carried out using 2× Taq PCR MasterMix II (TIANGEN Biotech Co., Ltd., Beijing, China). Primer sequences for each assay are detailed in Supplementary Table 2. Amplification products were resolved by agarose gel electrophoresis and observed using the ChemiDoc MP Imaging System (Bio-Rad Laboratories, Hercules, CA, USA). Products of the expected size were subsequently sequenced using the Sanger method on an ABI Prism 3730 Genetic Analyzer (Applied Biosystems, Foster City, CA, USA). Resulting sequences were assembled using the SeqMan software (version 7.1.0, DNASTAR, Madison, WI, USA), and genus-level taxonomic identification was performed via BLAST alignment against the NCBI GenBank database (National Center for Biotechnology Information, Bethesda, MD, USA).

### 2.7 Statistical analysis

Infection prevalence or seroprevalence was defined as the proportion of samples tested positive for nucleic acids or antibodies relative to the total number of samples tested. Coinfection prevalence, or co-seropositive prevalence, was calculated as the proportion of samples simultaneously positive for two or more pathogens. In calculating coinfection prevalence, all occurrences of a specific pathogen combination were included regardless of exclusivity. Group comparisons were conducted using the chi-square (χ^2^) test or Fisher's exact test. Univariate and multiple logistic regression models were applied to evaluate pathogen–pathogen associations, with odds ratios (ORs) and corresponding 95% confidence intervals (CIs) estimated. For multiple regression models evaluating associations between pathogen pairs, each pathogen was alternately designated as the exposure and the outcome. Adjustment for potential confounders was performed according to sample type: sex, age, occupation, and tick-bite history in community residents; sex, age, and occupation in SFTS patients; species, life stage, and parasitic phase in ticks; and species, sex, and capture type in small mammals, respectively. The identified associations should be interpreted as epidemiological associations based on co-occurrence patterns and do not imply biological or mechanistic interactions between pathogens. At the township level, Spearman's rank correlation was used to examine associations of infection and coinfection among community residents, ticks, and small mammals, using data from the six townships from which community residents were recruited (Supplementary Table 3), and correlation coefficients (rho) with 95% CIs were calculated. A *P*-value <0.05 was defined as statistically significant. All statistical analyses and data visualizations were conducted using R software, version 4.4.1.

## 3 Results

### 3.1 Characteristics and prevalence of four samples

Among 485 community residents, the majority were aged ≥60 years (72.0%), female (54.4%), and engaged in farming (73.6%). The IgG seroprevalence for *Anaplasma* spp., *Bartonella* spp., *Coxiella* spp., *Ehrlichia* spp. and SFGR was 18.6%, 10.3%, 12.8%, 11.8%, and 13.8%, respectively. Among 90 SFTS patients, most were aged ≥60 years (77.9%), male (58.9%), and engaged in farming (85.6%). For the five TBPs of *Anaplasma* spp., *Bartonella* spp., *Coxiella* spp., *Ehrlichia* spp. and SFGR, IgG seroprevalence was 23.3%, 23.3%, 15.6%, 16.7%, and 16.7%, while IgM seroprevalence was 8.9%, 3.3%, 5.6%, 4.4%, and 8.9%, respectively. A total of 1081 ticks were collected, predominantly *Haemaphysalis longicornis* (69.2%), followed by *Haemaphysalis hystricis* (8.5%). Adults (58.7%), and feeding ticks (53.0%) accounted for the majority of collected ticks. The prevalence of *Anaplasma* spp., *Bartonella* spp., *Coxiella* spp., *Ehrlichia* spp. and SFGR was 10.9%, 0.9%, 0.2%, 9.3% and 17.0%, respectively. Among 471 captured small mammals, *Apodemus agrarius* (26.3%) and *Rattus norvegicus* (20.4%) were the dominant species, while males (51.2%) and field-captured individuals (76.0%) predominated overall. The prevalence of *Anaplasma* spp., *Bartonella* spp., *Coxiella* spp., *Ehrlichia* spp. and SFGR was 9.3%, 10.8%, 3.0%, 2.8%, and 1.9%, respectively. Prevalence of the five TBPs by characteristics are shown in Table 1.

**Table 1:** Demographic characteristics and infection prevalence/seroprevalence of four sample types.

	Total	*Anaplasma* spp.	*Bartonella* spp.	*Coxiella* spp.	*Ehrlichia* spp.	SFGR
Community residents	485	90 (18.6)	50 (10.3)	62 (12.8)	57 (11.8)	67 (13.8)
Age group, years						
<60	136 (28.0)	18 (13.2)	13 (9.6)	14 (10.3)	14 (10.3)	8 (5.9)
≥60	349 (72.0)	72 (20.6)	37 (10.6)	48 (13.8)	43 (12.3)	59 (16.9)
Sex						
Male	221 (45.6)	49 (22.2)	25 (11.3)	22 (10.0)	24 (10.9)	38 (17.2)
Female	264 (54.4)	41 (15.5)	25 (9.5)	40 (15.2)	33 (12.5)	29 (11.0)
Occupation^a^						
Farmers	357 (73.6)	70 (19.6)	39 (10.9)	48 (13.4)	47 (13.2)	54 (15.1)
Others	108 (22.3)	16 (14.8)	9 (8.3)	11 (10.2)	7 (6.5)	10 (9.3)
Tick-bite						
Yes	243 (51.1)	58 (23.9)	30 (12.3)	32 (13.2)	39 (16.0)	42 (17.3)
No	242 (49.9)	32 (13.2)	20 (8.3)	30 (12.4)	18 (7.4)	25 (10.3)

SFTS patients-IgG	90	21 (23.3)	21 (23.3)	14 (15.6)	15 (16.7)	15 (16.7)
Age group, years						
<60	20 (22.2)	6 (30.0)	1 (5.0)	3 (15.0)	4 (20.0)	3 (15.0)
≥60	70 (77.8)	15 (21.4)	20 (28.6)	11 (15.7)	11 (15.7)	12 (17.1)
Sex						
Male	53 (58.9)	14 (26.4)	9 (17.0)	8 (15.1)	8 (15.1)	11 (20.8)
Female	37 (41.1)	7 (18.9)	12 (32.4)	6 (16.2)	7 (18.9)	4 (10.8)
Occupation						
Farmers	77 (85.6)	19 (24.7)	20 (26.0)	12 (15.6)	14 (18.2)	14 (18.2)
Others	13 (14.4)	2 (15.4)	1 (7.7)	2 (15.4)	1 (7.7)	1 (7.7)

SFTS patients-IgM	90	8 (8.9)	3 (3.3)	5 (5.6)	4 (4.4)	8 (8.9)
Age group, years						
<60	20 (22.2)	3 (15.0)	1 (5.0)	0 (0.0)	0 (0.0)	2 (10.0)
≥60	70 (77.8)	5 (7.1)	2 (2.9)	5 (7.1)	4 (5.7)	6 (8.6)
Sex						
Male	53 (58.9)	6 (11.3)	2 (3.8)	3 (5.7)	2 (3.8)	6 (11.3)
Female	37 (41.1)	2 (5.4)	1 (2.7)	2 (5.4)	2 (5.4)	2 (5.4)
Occupation						
Farmers	77 (85.6)	8 (10.4)	2 (2.6)	5 (6.5)	4 (5.2)	8 (10.4)
Others	13 (14.4)	0 (0.0)	1 (7.7)	0 (0.0)	0 (0.0)	0 (0.0)

Ticks	1081	118 (10.9)	10 (0.9)	2 (0.2)	100 (9.3)	184 (17.0)
Species						
*H. longicornis*	748 (69.2)	70 (9.4)	1 (0.1)	1 (0.1)	52 (7.0)	113 (15.1)
*H. hystricis*	92 (8.5)	31 (33.7)	2 (2.2)	0 (0.0)	11 (12.0)	11 (12.0)
*R. haemaphysaloides*	87 (8.1)	8 (9.2)	5 (5.7)	0 (0.0)	3 (3.4)	28 (32.2)
*R. sanguineus*	76 (7.0)	3 (3.9)	0 (0.0)	1 (1.3)	11 (14.5)	2 (2.6)
*R. microplus*	57 (5.3)	5 (8.8)	2 (3.5)	0 (0.0)	21 (36.8)	28 (49.1)
*I. sinensis*	20 (1.8)	1 (5.0)	0 (0.0)	0 (0.0)	2 (10.0)	2 (10.0)
*H. formosensis*	1 (0.1)	0 (0.0)	0 (0.0)	0 (0.0)	0 (0.0)	0 (0.0)
Life stages						
Larve	2 (0.2)	1 (50.0)	0 (0.0)	0 (0.0)	0 (0.0)	0 (0.0)
Nymph	444 (41.1)	56 (12.6)	1 (0.2)	0 (0.0)	23 (5.2)	54 (12.2)
Adult	635 (58.7)	61 (9.6)	9 (1.4)	2 (0.3)	77 (12.1)	130 (20.5)
Parasitic phase^a^						
Feeding tick	573 (53.0)	99 (17.3)	9 (1.6)	2 (0.3)	69 (12.0)	108 (18.8)
Questing tick	376 (34.8)	18 (4.8)	1 (0.3)	0 (0.0)	16 (4.3)	33 (8.8)

Small mammals	471	44 (9.3)	51 (10.8)	14 (3.0)	13 (2.8)	9 (1.9)
Species						
*A. agrarius*	124 (26.3)	3 (2.4)	43 (34.7)	1 (0.8)	1 (0.8)	2 (1.6)
*R. norvegicus*	96 (20.4)	7 (7.3)	1 (1.0)	3 (3.1)	2 (2.1)	2 (2.1)
*S. murinus*	52 (11.0)	0 (0.0)	0 (0.0)	0 (0.0)	0 (0.0)	0 (0.0)
*N. coninga*	48 (10.2)	8 (16.7)	0 (0.0)	0 (0.0)	5 (10.4)	2 (4.2)
*N. niviventer*	43 (9.2)	10 (23.3)	1 (2.3)	3 (7.0)	5 (11.6)	1 (2.3)
*R. losea*	32 (6.8)	3 (9.4)	4 (12.5)	0 (0.0)	0 (0.0)	0 (0.0)
*N. coxingi*	23 (4.9)	9 (39.1)	0 (0.0)	5 (21.7)	0 (0.0)	1 (4.3)
*M. musculus*	22 (4.7)	0 (0.0)	1 (4.5)	1 (4.5)	0 (0.0)	1 (4.5)
*R. flavipectus*	19 (4.0)	0 (0.0)	1 (5.3)	0 (0.0)	0 (0.0)	0 (0.0)
*B. bowersi*	9 (1.9)	2 (22.2)	0 (0.0)	1 (11.1)	0 (0.0)	0 (0.0)
*M. minutus*	2 (0.4)	2 (100.0)	0 (0.0)	0 (0.0)	0 (0.0)	0 (0.0)
*N. confucianus*	1 (0.2)	0 (0.0)	0 (0.0)	0 (0.0)	0 (0.0)	0 (0.0)
Sex						
Male	241 (51.2)	32 (13.3)	32 (13.3)	7 (2.9)	8 (3.3)	7 (2.9)
Female	230 (48.8)	12 (5.2)	19 (8.3)	7 (3.0)	5 (2.2)	2 (0.9)
Capture types^a^						
Field-captured	358 (76.0)	36 (10.1)	49 (13.7)	13 (3.6)	11 (3.1)	9 (2.5)
Peri-domestic captured	82 (17.4)	8 (9.8)	2 (2.4)	1 (1.2)	2 (2.4)	0 (0.0)

Values are n (%). ^a^: Presence of missing values. Abbreviations: *A. agrarius*, *Apodemus agrarius*; *B. bowersi*, *Berylmys bowersi*; *H. formosensis*, *Haemaphysalis formosensis*; *H. hystricis*, *Haemaphysalis hystricis*; *H. longicornis*, *Haemaphysalis longicornis*; *I. sinensis*, *Ixodes sinensis*; *M. minutus*, *Micromys minutus*; *M. musculus*, *Mus musculus*; *N. confucianus*, *Niviventer confucianus*; *N. coninga*, *Niviventer coninga*; *N. coxingi*, *Niviventer coxingi*; *N. niviventer*, *Niviventer niviventer*; SFGR, spotted fever group rickettsiae; *S. murinus*, *Suncus murinus*; *R. flavipectus*, *Rattus flavipectus*; *R. haemaphysaloides*, *Rhipicephalus haemaphysaloides*; *R. losea*, *Rattus losea*; *R. microplus*, *Rhipicephalus microplus*; *R. norvegicus*, *Rattus norvegicus*; *R. sanguineus*, *Rhipicephalus sanguineus*.

Notably, IgG seroprevalence for *Anaplasma* spp., *Ehrlichia* spp. and SFGR was significantly higher in the tick-bite group than in the no tick-bite group (*P* = 0.004, *P* = 0.005, and *P* = 0.037, respectively), while no significant differences were observed between the tick-bite group and SFTS patients (Supplementary Table 2). For *Bartonella* spp., a significant difference in the IgG seroprevalence was observed only between the tick-bite group and SFTS patients (12.3% vs. 23.3%; *P* = 0.021), while *Coxiella* spp. showed no significant differences within any groups (Supplementary Table 4).

### 3.2 Coinfection and Co-seropositivity patterns among four samples

Coinfections were observed across all sample types, but with varying frequencies. In community residents, multiple co-seropositivity patterns were identified, with the highest prevalence observed for *Anaplasma* spp./*Ehrlichia* spp. (6.6%), followed by *Anaplasma* spp./SFGR (3.7%) and *Ehrlichia* spp./SFGR (3.3%). Among SFTS patients, IgG co-seropositivity was more common, with the highest prevalence for *Anaplasma* spp./*Ehrlichia* spp. (8.9%), and five additional combinations exceeding 4%. IgM co-serepositivity in SFTS patients was uncommon, with only four patterns identified, each with a prevalence of 1.1%. In ticks and small mammals, coinfection events were sporadic, with the highest prevalence recorded for *Ehrlichia* spp./SFGR (2.4%) in ticks and *Anaplasma* spp./*Coxiella* spp. (1.3%) in small mammals. The prevalence of all coinfection and co-seropositivity patterns is summarized in Fig. 1.

**Fig. 1:**
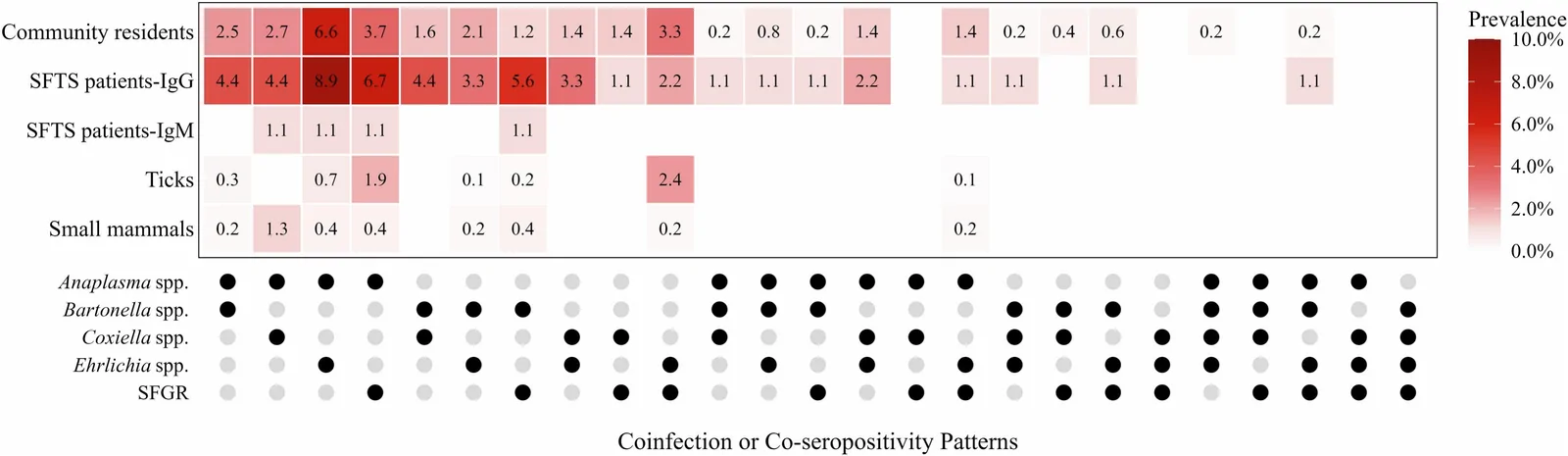
Heatmap of tick-borne pathogen coinfection patterns. Numbers indicate infection prevalence or co-seropositive prevalence. Color intensity represents the magnitude of prevalence. Black dots indicate the presence of a pathogen in the pattern, while gray dots indicate its absence.

Notably, significant differences in IgG co-seroprevalence were observed only between the tick-bite and no tick-bite groups (*P* < 0.001). Specifically, the co-seroprevalence of *Anaplasma* spp./SFGR or *Ehrlichia* spp./SFGR was significantly higher in the tick-bite group compared with the no tick-bite group (*P* = 0.031 and *P* = 0.005, respectively), as shown in Supplementary Table 5.

### 3.3 Pathogen-pathogen associations in human samples

In community residents, significant associations were observed for *Anaplasma* spp./*Ehrlichia* spp. (cOR = 8.17, 95% CI: 4.52–14.75, *P* < 0.001) and *Ehrlichia* spp./SFGR (cOR = 2.88, 95% CI: 1.51–5.51, *P* = 0.001). Among SFTS patients, an association of *Anaplasma* spp./*Ehrlichia* spp. was identified for IgG (cOR = 5.45, 95% CI: 1.68–17.69, *P* = 0.005), whereas no significant associations were observed for IgM. After Adjusting for potential confounders, the associations remained significant for *Anaplasma* spp./*Ehrlichia* spp. (aOR1 = 8.46, 95% CI: 4.50–15.90, *P* < 0.001; aOR2 = 8.52, 95% CI: 4.52–16.05, *P* < 0.001) and *Ehrlichia* spp./SFGR (aOR1 = 2.51, 95% CI: 1.25–5.05, *P* = 0.010; aOR2 = 2.51, 95% CI: 1.25–5.04, *P* = 0.010) in community residents, and for the IgG association of *Anaplasma* spp./*Ehrlichia* spp. in SFTS patients (aOR1 = 5.47, 95% CI: 1.62–18.44, *P* = 0.006; aOR2 = 5.44, 95% CI: 1.61–18.36, *P* = 0.006). Detailed results are shown in Table 2.

**Table 2:** Pathogen–Pathogen associations across humans, ticks, and small mammals

	cOR (95%CI)	*P*-value	aOR1 (95%CI)	*P*-value	aOR2 (95%CI)	*P*-value
Community residents						
*Anaplasma* spp./*Bartonella* spp.	1.45 (0.72-2.89)	0.298	1.24 (0.60-2.59)	0.560	1.24 (0.60-2.58)	0.568
*Anaplasma* spp.*/Coxiella* spp.	1.19 (0.62-2.31)	0.601	0.90 (0.43-1.89)	0.784	0.90 (0.43-1.89)	0.782
*Anaplasma* spp./*Ehrlichia* spp.	8.17 (4.52-14.76)	**<0.001**	8.46 (4.50-15.90)	**<0.001**	8.52 (4.52-16.05)	**<0.001**
*Anaplasma* spp./SFGR	1.77 (0.97-3.21)	0.062	1.54 (0.83-2.88)	0.174	1.52 (0.81-2.85)	0.194
*Bartonella* spp./*Coxiella* spp.	1.34 (0.60-3.01)	0.473	1.24 (0.53-2.94)	0.621	1.24 (0.52-2.92)	0.631
*Bartonella* spp./*Ehrlichia* spp.	2.06 (0.97-4.40)	0.060	1.78 (0.80-3.97)	0.161	1.77 (0.79-3.96)	0.165
*Bartonella* spp./SFGR	0.84 (0.34-2.05)	0.695	0.78 (0.31-1.96)	0.601	0.79 (0.31-1.98)	0.609
*Coxiella* spp./*Ehrlichia* spp.	0.95 (0.41-2.20)	0.904	0.79 (0.32-1.96)	0.604	0.80 (0.32-1.98)	0.625
*Coxiella* spp./SFGR	0.77 (0.33-1.77)	0.538	0.80 (0.34-1.89)	0.616	0.82 (0.35-1.94)	0.655
*Ehrlichia* spp./SFGR	2.88 (1.51-5.51)	**0.001**	2.51 (1.25-5.05)	**0.010**	2.51 (1.25-5.04)	**0.010**
SFTS patients-IgG						
*Anaplasma* spp./*Bartonella* spp.	0.72 (0.21-2.44)	0.597	0.80 (0.22-2.85)	0.727	0.85 (0.24-3.08)	0.810
*Anaplasma* spp.*/Coxiella* spp.	1.39 (0.39-4.99)	0.615	1.40 (0.38-5.13)	0.611	1.42 (0.39-5.19)	0.597
*Anaplasma* spp./*Ehrlichia* spp.	5.45 (1.68-17.69)	**0.005**	5.47 (1.62-18.44)	**0.006**	5.44 (1.61-18.36)	**0.006**
*Anaplasma* spp./SFGR	2.67 (0.82-8.66)	0.103	2.45 (0.72-8.22)	0.148	2.49 (0.74-8.35)	0.139
*Bartonella* spp./*Coxiella* spp.	1.39 (0.39-4.99)	0.615	1.59 (0.41-6.13)	0.502	1.40 (0.36-5.34)	0.627
*Bartonella* spp./*Ehrlichia* spp.	0.79 (0.20-3.12)	0.739	0.82 (0.20-3.39)	0.781	0.74 (0.18-3.11)	0.684
*Bartonella* spp./SFGR	1.84 (0.55-6.17)	0.321	2.07 (0.56-7.62)	0.272	2.10 (0.58-7.71)	0.261
*Coxiella* spp./*Ehrlichia* spp.	1.45 (0.35-6.00)	0.604	1.46 (0.35-6.12)	0.603	1.43 (0.34-6.02)	0.629
*Coxiella* spp./SFGR	0.34 (0.04-2.82)	0.318	0.34 (0.04-2.84)	0.317	0.33 (0.04-2.77)	0.305
*Ehrlichia* spp./SFGR	0.73 (0.15-3.65)	0.705	0.70 (0.14-3.61)	0.673	0.69 (0.13-3.53)	0.653
SFTS patients-IgM						
*Anaplasma* spp./*Coxiella* spp.	2.79 (0.27-28.45)	0.388	3.23 (0.28-37.00)	0.346	3.30 (0.29-37.20)	0.334
*Anaplasma* spp./*Ehrlichia* spp.	3.76 (0.34-41.12)	0.278	4.83 (0.39-60.11)	0.221	4.65 (0.39-56.08)	0.226
*Anaplasma* spp./SFGR	1.53 (0.16-14.29)	0.709	0.97 (0.10-9.83)	0.979	0.98 (0.10-10.00)	0.987
*Bartonella* spp./SFGR	5.71 (0.46-71.14)	0.176	9.85 (0.52-186.80)	0.128	12.66 (0.61-260.91)	0.100
Ticks						
*Anaplasma* spp./*Bartonella* spp.	3.56 (0.91-13.97)	0.068	3.59 (0.78-16.50)	0.101	3.32 (0.73-15.02)	0.120
*Anaplasma* spp./*Ehrlichia* spp.	0.69 (0.33-1.46)	0.329	0.66 (0.29-1.48)	0.316	0.64 (0.29-1.43)	0.276
*Anaplasma* spp./SFGR	1.06 (0.64-1.75)	0.812	1.13 (0.65-1.98)	0.660	1.15 (0.65-2.02)	0.631
*Bartonella* spp./*Ehrlichia* spp.	1.09 (0.14-8.70)	0.935	0.74 (0.08-7.27)	0.795	0.76 (0.08-7.34)	0.809
*Bartonella* spp./SFGR	1.22 (0.26-5.80)	0.802	0.53 (0.10-2.78)	0.453	0.58 (0.11-2.95)	0.510
*Ehrlichia* spp./SFGR	1.83 (1.13-2.95)	**0.013**	1.29 (0.75-2.24)	0.356	1.34 (0.78-2.31)	0.287
Small mammals						
*Anaplasma* spp./*Bartonella* spp.	0.18 (0.02-1.30)	0.089	0.35 (0.04-2.93)	0.332	0.33 (0.04-2.77)	0.304
*Anaplasma* spp./*Coxiella* spp.	8.27 (2.73-25.08)	**<0.001**	6.03 (1.79-20.34)	**0.004**	5.67 (1.67-19.29)	**0.005**
*Anaplasma* spp./*Ehrlichia* spp.	1.80 (0.39-8.40)	0.454	0.80 (0.16-4.11)	0.793	0.90 (0.18-4.62)	0.903
*Anaplasma* spp./SFGR	2.86 (0.58-14.20)	0.199	1.87 (0.35-10.05)	0.462	1.68 (0.31-9.10)	0.550
*Bartonella* spp./*Ehrlichia* spp.	0.68 (0.09-5.34)	0.714	3.47 (0.21-56.08)	0.380	3.08 (0.19-50.83)	0.431
*Bartonella* spp./SFGR	2.41 (0.49-11.92)	0.281	4.78 (0.50-46.06)	0.176	5.12 (0.54-48.72)	0.155
*Ehrlichia* spp./SFGR	4.69 (0.54-40.50)	0.160	3.05 (0.30-31.42)	0.348	3.33 (0.30-37.60)	0.331

cOR: Crude odds ratio. aOR1: Adjusted odds ratio with the first pathogen in the pair as the outcome and the second as the exposure. aOR2: Adjusted odds ratio with the second pathogen in the pair as the outcome and the first as the exposure. Abbreviations: SFGR: spotted fever group rickettsiae; SFTS: severe fever with thrombocytopenia syndrome.

### 3.4 Pathogen-pathogen associations in ticks and small mammals

In ticks, a significant association was observed for *Ehrlichia* spp./SFGR (cOR = 1.83, 95% CI: 1.13–2.95, *P* = 0.013); however, this association was no longer statistically significant after adjustment. In small mammals, a significant association between *Anaplasma* spp. and *Coxiella* spp. was observed (cOR = 8.27, 95% CI: 2.73–25.08, *P* < 0.001), and the association remained robust after adjustment (aOR1 = 6.04, 95% CI: 1.79–20.40, *P* = 0.004; aOR2 = 5.71, 95% CI: 1.68–19.42, *P* = 0.005). Detailed results are shown in Table 2. Given that species was statistically significant in the adjusted model, we further stratified the analysis by small mammal species. The results indicated that the association persisted exclusively in *Rattus norvegicus*, with strong associations maintained even after adjustment for sex and capture types (aOR1 = 85.86, 95% CI: 6.74–12,425.52, *P* < 0.001; aOR2 = 60.56, 95% CI: 5.98–6263.92, *P* < 0.001).

### 3.5 Correlation of pathogens between community residents, ticks, and small mammals

We further evaluated correlations in pathogen prevalence among community residents, ticks, and small mammals. Specifically, SFGR exhibited a significant correlation between ticks and community residents (rho = 0.89, *P* = 0.019), whereas *Bartonella* spp. showed a significant correlation between small mammals and community residents (rho = 0.85, *P* = 0.034). In terms of coinfections, *Anaplasma* spp.*/Ehrlichia* spp. (rho = 0.88, *P* = 0.021) and *Anaplasma* spp./SFGR (rho = 0.81, *P* = 0.050) were significantly correlated between ticks and community residents. However, no significant ecological associations were observed between ticks and small mammals. The correlations are presented in Fig. 2.

**Fig. 2:**
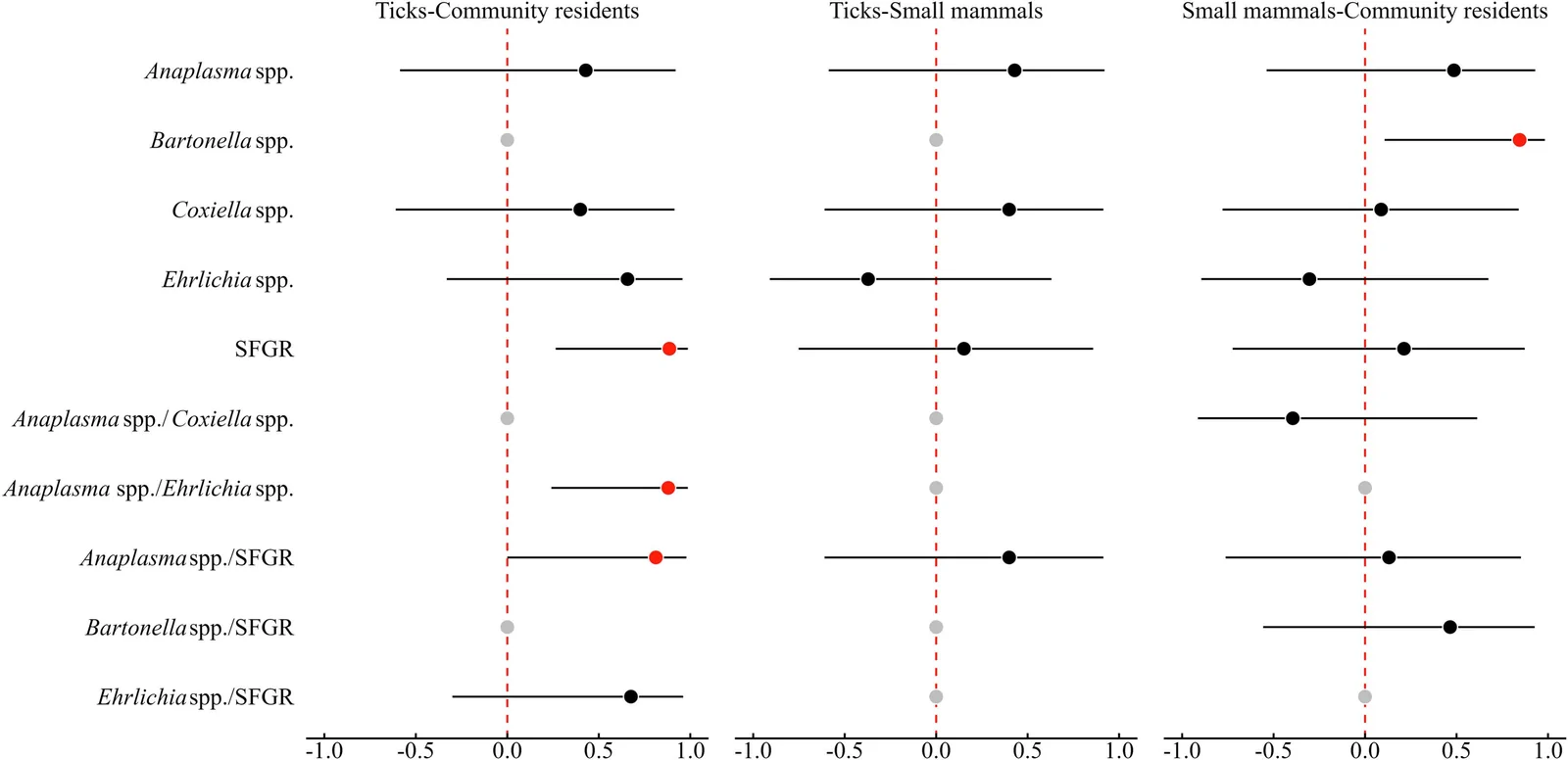
Forest plot of correlations in tick-borne pathogen infections and coinfections. Points represent rho, red indicates *P*-value < 0.05, and lines represent 95% CIs. (For interpretation of the references to color in this figure legend, the reader is referred to the web version of this article.)

## 4 Discussion

In this study, we systematically investigated five major TBPs in vectors and hosts within the same environment using a One Health approach. All five TBPs were detected across the four sample types, although their prevalence varied substantially. Diverse co-seropositivity patterns were observed in IgG detected from human samples, while coinfection patterns in ticks and small mammals were comparatively rare. Significant pathogen-pathogen associations were identified, specifically *Anaplasma* spp./*Ehrlichia* spp. and *Ehrlichia* spp./SFGR in human IgG, and *Anaplasma* spp./ *Coxiella* spp. in small mammals. In addition, cross-host correlations in prevalence were observed: SFGR prevalence in community residents was positively associated with its prevalence in ticks, while *Bartonella* spp. prevalence in community residents was correlated with that in small mammals. Furthermore, *Anaplasma* spp./*Ehrlichia* spp. and *Anaplasma* spp./SFGR showed significant associations between ticks and community residents.

Tick-borne diseases are primarily transmitted by ticks and are characterized by high transmissibility and wide geographic distribution [30]. Among these diseases, SFTS is the most frequently reported tick-borne disease in China [30]. Zhejiang Province not only has a relatively high incidence of SFTS but also a case fatality rate that exceeds the national average [31], and Taizhou City has reported the highest number of cases in the province since 2017 [27]. Our findings indicate that in this epidemic area of SFTS, multiple TBPs are circulating and show ecological associations across hosts, with important implications for human health. In addition, we detected IgM antibodies against other TBPs in SFTS patients, suggesting recent infections or possible coinfections, although the prevalence was relatively low. In China, reports of coinfections between SFTS and other TBPs remain scarce [32], [33], [34], but previous studies have suggested that bacterial coinfections may increase mortality risk in SFTS patients [35]. Therefore, clinicians should remain vigilant for the potential presence of other TBPs in SFTS patients and should consider targeted diagnostic testing and treatment.

IgG antibodies induced by TBPs can persist for extended periods in the host [36], [37], which may account for the broader diversity of IgG co-seropositivity patterns observed in humans, in contrast to the comparatively limited coinfection combinations detected in ticks and small mammals. This finding suggests that IgG co-seropositivity in humans is more likely to reflect repeated or long-term exposures, rather than a single tick bite involving multiple pathogens. An important question is whether the IgG antibodies against all five pathogens detected in humans are primarily acquired through tick bites. To address this question, we compared IgG seropositivity among the tick-bite group, the no tick-bite group, and SFTS patients. Seropositivity for *Anaplasma* spp., *Ehrlichia* spp., and SFGR differed only between the tick-bite and no tick-bite groups, whereas *Bartonella* spp. showed differences between the tick-bite group and SFTS patients. These findings indicate that different TBPs likely follow distinct exposure pathways, which should be carefully considered when interpreting coinfection patterns. It should also be noted that tick-bite history relies on participants' recall, which may lead to exposure misclassification. This may affect the accuracy of group comparisons and potentially result in underestimation or overestimation of differences in seropositivity between groups.

*Anaplasma* spp., *Ehrlichia* spp., and SFGR are members of the order Rickettsiales [38]. These pathogen groups include agents responsible for human anaplasmosis, human ehrlichiosis, and spotted fever rickettsioses, respectively [39]. These pathogens have been detected in a wide range of tick species worldwide. [40], [41], [42]. In China, *H. longicornis* is one of the dominant tick species and has been frequently associated with the circulation and transmission of these pathogens [30], [39]. In Taizhou City, *H. longicornis* constitutes the dominant tick species (69.2%). In our study, the prevalence of *Anaplasma* spp., *Ehrlichia* spp., and SFGR within this species was 9.4%, 7.0%, and 15.1%, respectively, suggesting that *H. longicornis* may act as a common transmission vector for these three pathogens. Notably, *H. longicornis* is also the principal vector of SFTS virus [27], highlighting the importance of monitoring potential coinfections with these TBPs in SFTS patients. In community residents, we observed significant associations in IgG antibodies to *Anaplasma* spp./*Ehrlichia* spp. and *Ehrlichia* spp./SFGR, whereas such patterns were not apparent in individual ticks. This finding suggests that the associations observed in community residents may result from shared exposure pathways and repeated or long-term exposures. Moreover, we found that the prevalence of IgG co-seropositivity for *Anaplasma* spp./*Ehrlichia* spp. and *Anaplasma* spp./SFGR in humans was correlated with the coinfection prevalence observed in ticks, which may be attributable to the high infection prevalence of these three pathogens in ticks. This results in occasional coinfections in ticks, while in humans, it manifests as antibody associations driven by shared exposure pathways.

*Bartonella* spp. is transmissible through multiple routes, including hematophagous arthropods (such as cat fleas, ticks, and sandflies) as well as animal scratches or bites, and is capable of causing a range of diseases [43]. Although *Bartonella* spp. has been detected in ticks in several studies [44], [45], there is no conclusive evidence that ticks can transmit *Bartonella* spp. to humans [46], which is consistent with our findings. In contrast, previous studies have demonstrated that rodents are important reservoirs of *Bartonella* spp. and that transmission may occur through fleas carried by rodents [47], [48]. In the present study, we identified a correlation between the prevalence of *Bartonella* spp. in community residents and that in small mammals. This suggests that in Taizhou City, *Bartonella* spp. transmission is likely primarily associated with small mammals, occurring either through direct contact or via bites from ectoparasites such as fleas or lice. Notably, *A. agrarius*, with a prevalence of 34.7%, may act as a key reservoir host, playing an important role in the natural cycle of *Bartonella* spp.

Detected *Coxiella* spp. may include pathogenic *Coxiella burnetii* as well as *Coxiella*-like endosymbionts (CLEs), whose pathogenic potential in humans remains unclear [49], [50]. In the present study, the prevalence of *Coxiella* spp. was low in ticks (0.2%) but higher in small mammals (3.0%), suggesting that small mammals may contribute to the ecological maintenance of Coxiella spp. in this SFTS endemic area [51]. We also observed a significant association between *Anaplasma* spp. and *Coxiella* spp. in small mammals, which may reflect shared exposure patterns or host susceptibility differences. However, species-level discrimination could not be fully achieved due to limited molecular evidence. Further studies are needed to clarify the taxonomic identity and epidemiological significance of the detected *Coxiella* spp. The *Coxiella* spp. sequences generated in this study have been deposited in the NCBI GenBank database (PZ595279-PZ595294).

Several limitations have to be considered. First, as a cross-sectional study, sample collection was not conducted simultaneously, and thus our findings are more likely to reflect ecological concordance rather than provide direct evidence of transmission. Second, information on tick bite history relied on participant recall, which may introduce recall bias and affect the accuracy of exposure information and the reliability of the analytical results. Third, the analysis was restricted to the genus level and could not distinguish between pathogenic and non-pathogenic species, mainly because the relatively low coinfection prevalence would have limited the stability of the estimates under further stratification. Fourth, the relatively small number of SFTS cases may have contributed to the instability of certain OR estimates. Finally, IFA-based serological testing may be subject to cross-reactivity, potentially leading to overestimation of true prevalence.

## 5 Conclusion

This study demonstrates that multiple TBPs co-occurrence in this SFTS endemic area, with circulation involving ticks, small mammals, and humans. Transmission ways appear to differ among pathogens: *Anaplasma* spp., *Ehrlichia* spp., and SFGR are primarily exposured by ticks, whereas *Bartonella* spp. is more likely associated with small mammals. The observed serological and ecological patterns indicate that IgG co-seropositivity in humans is more likely to reflect repeated or long-term exposures rather than a single tick bite carrying multiple pathogens. These findings suggest that prevention efforts should account for human activity patterns and the role of small mammals in sustaining pathogen circulation, rather than focusing solely on tick control. Adopting a One Health approach facilitates the integration of data from humans, animals, and vectors, enhancing understanding of pathogen transmission and guiding the development of more targeted prevention and control strategies. Future studies should include livestock and larger wild animals and apply molecular approaches to better characterize pathogen associations across humans, animals, and vectors, thereby providing a more comprehensive understanding of their circulation within a One Health framework.

## CRediT authorship contribution statement

**Zhiyi Zhang**: Writing – original draft, Methodology, Investigation, Formal analysis, Data curation, Conceptualization. **Bingzhi Chen**: Methodology, Formal analysis, Data curation. **Yishuang Zhao**: Methodology, Data curation. **Xiang Zheng**: Data curation, Investigation. **Qing Gao**: Data curation, Investigation. **Hongbiao Liang**: Data curation, Investigation. **Haijiang Lin**: Writing – review & editing, Supervision, Conceptualization. **Na He**: Writing – review & editing, Supervision, Funding acquisition.

## Funding

This study was sponsored by 10.13039/501100003347Fudan University (Grant No. DGF201004, DGF201006, and DGF201016), Taizhou City Foundation for Talents (grant no. TZ2022-2) and the Zhejiang Province Leading Earth Goose Program (2025C02186).

## Declaration of competing interest

The authors declare that they have no known competing financial interests or personal relationships that could have appeared to influence the work reported in this paper.

## Data Availability

Data will be made available on request.

## Appendix A Supplementary data

Supplementary material: supplementary tables providing additional information on the kits used, sample characteristics, cross-host analyses, and comparisons of TBP infections among study populations.
