# Supplementary material for: Coinfection patterns and associations of tick-borne pathogens in natural foci of severe fever with thrombocytopenia syndrome: A One Health approach
Source: One Health. 2026 Sep 17;23:101543. doi: 10.1016/j.onehlt.2026.101543 (PMC13737371; doi:10.1016/j.onehlt.2026.101543)
Supplement: Supplementary file 1 [file mmc1.docx]

Supplementary Table 1 Specifications and interpretation criteria for kits used

| Genus | Kit | Cat. no. | Subtype | Interpretation criteria |
| --- | --- | --- | --- | --- |
| *Anaplasma* spp. | Anaplasma phagocytophilum and Ehrlichia chaffeensis MIF  IgG Antibody Kit | E21X-120 | *Anaplasma*  *phagocytophilum* | Serum titers from 1:64 dilution or greater were considered positive. |
| *Anaplasma* spp. | Ehrlichia chaffeensis and  Anaplasma phagocytophilum IFA IgM Antibody Kit | E21M-120 | *Anaplasma*  *phagocytophilum* | Serum titers from 1:64 dilution or greater were considered positive. |
| *Bartonella* spp. | Bartonella henselae IFA  Human IgG Antibody Kit | BHG-120 | *Bartonella henselae* | Serum titers from 1:64 dilution or greater were considered positive. |
| *Bartonella* spp. | Bartonella henselae IFA  Human IgM Antibody Kit | BHM-120 | *Bartonella henselae* | Serum titers from 1:64 dilution or greater were considered positive. |
| *Coxiella* spp. | Q-fever IFA  IgG Antibody Kit | QG-120 | *Coxiella burnetii* | Seropositivity was defined by the concurrent presence of IgG titers ≥1:16 against both phase I and phase II antigens, with phase I titers exceeding those of phase II. |
| *Coxiella* spp. | Q-fever IFA  IgM Antibody Kit | QM-120 | *Coxiella burnetii* | IgM titers of 1:16 or higher against either phase I or phase II were considered positive. |
| *Ehrlichia* spp. | Anaplasma phagocytophilum and Ehrlichia chaffeensis MIF  IgG Antibody Kit | E21X-120 | *Ehrlichia chaffeensis* | Serum titers from 1:64 dilution or greater were considered positive. |
| *Ehrlichia* spp. | Ehrlichia chaffeensis and  Anaplasma phagocytophilum IFA IgM Antibody Kit | E21M-120 | *Ehrlichia chaffeensis* | Serum titers from 1:64 dilution or greater were considered positive. |
| SFGR | Rickettsia rickettsii IFA  IgG Antibody Kit | RRG-120 | *Rickettsia rickettsii* | Serum titers from 1:64 dilution or greater were considered positive. |
| SFGR | Rickettsia Screen  IFA IgM Antibody Kit | R20M-120 | *Rickettsia rickettsii* | Serum titers from 1:64 dilution or greater were considered positive. |

Supplementary Table 2 PCR primers and conditions used for TBPs

| Target agent | Target gene | Primer name | Prime Sequences 5’-3’ | Annealing (°C) | Amplicon size (bp) |
| --- | --- | --- | --- | --- | --- |
| *Anaplasma phagocytophilum* | 16S rRNA | Eh-out1 | TTGAGAGTTTGATCCTGGCTCAGAACG | 54 | 653 |
|  |  | Eh-out2 | CACCTCTACACTAGGAATTCCGCTATC |  |  |
|  |  | HGA1 | GTCGAACGGATTATTCTTTATAGCTTG | 55 | 389 |
|  |  | HGA2 | TATAGGTACCGTCATTATCTTCCCTAC |  |  |
| *Bartonella* spp. | gltA | Bart781 | GGGGACCAGCTCATGGTGG | 55 | 356 |
|  |  | Bart1137 | AATGCAAAAAGAACAGTAAACA |  |  |
| *Coxiella burnetii* | IS1111 | IS1111F1 | TACTGGGTGTTGATATTGC | 58 | 521 |
|  |  | IS1111R1 | CCGTTTCATCCGCGGTG |  |  |
|  |  | IS1111F2 | GTAAAGTGATCTACACGA | 53 | 297 |
|  |  | IS1111R2 | TTAACAGCGCTTGAACGT |  |  |
| *Ehrlichia chaffeensis* | 16S rRNA | Eh-out1 | TTGAGAGTTTGATCCTGGCTCAGAACG | 54 | 653 |
|  |  | Eh-out2 | CACCTCTACACTAGGAATTCCGCTATC |  |  |
|  |  | HME1 | CAATTGCTTATAACCTTTTGGTTATAAAT | 55 | 389 |
|  |  | HME2 | TATAGGTACCGTCATTATCTTCCCTAT |  |  |
| SFGR | ompA | Rr190.70p | ATGGCGAATATTTCTCCAAAA | 50 | 532 |
|  |  | Rr190.602n | AGTGCAGCATTCGCTCCCCCT |  |  |
|  |  | 190.70-38s1 | AAAACCGCTTTATTCACC | 50 | 346 |
|  |  | 190.602-384r | GGCAACAAGTTACCTCCT |  |  |

Supplementary Table 3 Sample composition of cross-host analysis

| Townships | Community residents  (n) | Ticks  (n) | Small mammals  (n) |
| --- | --- | --- | --- |
| Yongxi township | 144 | 88 | 54 |
| Tantou town | 36 | 107 | 59 |
| Dachen town | 94 | 65 | 31 |
| Baishuiyang town | 83 | 76 | 22 |
| Yongquan town | 93 | 60 | 31 |
| Anzhou subdistrict | 35 | 61 | 49 |

Supplementary Table 4 Comparison of TBP infections between community residents and SFTS patients

|  | Community residents | | SFTS patients  （n=90） | *P*-value^a^ | *P*-value^b^ |
| --- | --- | --- | --- | --- | --- |
|  | Tick-bite group（n=243） | No tick-bite group（n=242） |  |  |  |
| *Anaplasma* spp. |  |  |  | **0.004** | 1.000 |
| Positive | 58 (23.9) | 32 (13.2) | 21 (23.3) |  |  |
| Negative | 185 (76.1) | 210 (86.8) | 69 (76.7) |  |  |
| *Bartonella* spp. |  |  |  | 0.184 | **0.021** |
| Positive | 30 (12.3) | 20 (8.3) | 21 (23.3) |  |  |
| Negative | 213 (87.7) | 222 (91.7) | 69 (76.7) |  |  |
| *Coxiella* spp. |  |  |  | 0.906 | 0.703 |
| Positive | 32 (13.2) | 30 (12.4) | 14 (15.6) |  |  |
| Negative | 211 (86.8) | 212 (87.6) | 76 (84.4) |  |  |
| *Ehrlichia* spp. |  |  |  | **0.005** | 1.000 |
| Positive | 39 (16.0) | 18 (7.4) | 15 (16.7) |  |  |
| Negative | 204 (84.0) | 224 (92.6) | 75 (83.3) |  |  |
| SFGR |  |  |  | **0.037** | 1.000 |
| Positive | 42 (17.3) | 25 (10.3) | 15 (16.7) |  |  |
| Negative | 201 (82.7) | 217 (89.7) | 75 (83.3) |  |  |

Values are n (%). ^a^: compared between tick bite group and no tick bite group. ^b^: compared between tick bite group and SFTS patients. Abbreviations: SFGR: Spotted fever group rickettsiae.

Supplementary Table 5 Comparison of IgG co-seropositivity between community residents and SFTS patients

|  | Community residents | | SFTS patients  （n=90） | *P*-value^a^ | *P*-value^b^ |
| --- | --- | --- | --- | --- | --- |
|  | Tick-bite group（n=243） | No tick-bite group（n=242） |  |  |  |
| Co-seropositivity |  |  |  | **<0.001** | 0.281 |
| Yes | 57 (23.5) | 26 (10.7) | 27 (30.0) |  |  |
| No | 186 (76.5) | 216 (89.3) | 63 (70.0) |  |  |
| Number of co-seropositivity |  |  |  | **<0.001**^c^ | 0.688^c^ |
| 0 | 114 (46.9) | 150 (62.0) | 37 (41.1) |  |  |
| 1 | 72 (29.7) | 66 (27.3) | 26 (28.9) |  |  |
| 2 | 44 (18.1) | 19 (7.8) | 22 (24.5) |  |  |
| 3 | 11 (4.5) | 7 (2.9) | 4 (4.4) |  |  |
| 4 | 2 (0.8) | 0 (10.0) | 1 (1.1) |  |  |
| Co-seropositivity patterns |  |  |  |  |  |
| Two TBPs |  |  |  |  |  |
| *Anaplasma* spp./*Bartonella* spp. | 7 (2.9) | 5 (2.1) | 4 (4.4) | 0.776 | 0.497^c^ |
| *Anaplasma* spp./*Coxiella* spp. | 9 (3.7) | 4 (1.7) | 4 (4.4) | 0.264 | 0.754^c^ |
| *Anaplasma* spp./*Ehrlichia* spp. | 21 (8.6) | 11 (4.5) | 8 (8.9) | 0.102 | 1.000 |
| *Anaplasma* spp./SFGR | 14 (5.8) | 4 (1.7) | 6 (6.7) | **0.031** | 0.961 |
| *Bartonella* spp./*Coxiella* spp. | 3 (1.2) | 5 (2.1) | 4 (4.4) | 0.504^c^ | 0.089^c^ |
| *Bartonella* spp./*Ehrlichia* spp. | 7 (2.9) | 3 (1.2) | 3 (3.3) | 0.339^c^ | 0.733^c^ |
| *Bartonella* spp./SFGR | 5 (2.1) | 1 (0.4) | 5 (5.6) | 0.216^c^ | 0.141^c^ |
| *Coxiella* spp./*Ehrlichia* spp. | 4 (1.6) | 3 (1.2) | 3 (3.3) | 1.000^c^ | 0.393^c^ |
| *Coxiella* spp./SFGR | 5 (2.1) | 2 (0.8) | 1 (1.1) | 0.450^c^ | 1.000^c^ |
| *Ehrlichia* spp./SFGR | 14 (5.8) | 2 (0.8) | 2 (2.2) | **0.005** | 0.252^c^ |
| Three TBPs |  |  |  |  |  |
| *Anaplasma* spp./*Bartonella* spp./*Coxiella* spp. | 1 (0.4) | 0 (0.0) | 1 (1.1) | 1.000^c^ | 0.468^c^ |
| *Anaplasma* spp./*Bartonella* spp./*Ehrlichia* spp. | 2 (0.8) | 2 (0.8) | 1 (1.1) | 1.000^c^ | 1.000^c^ |
| *Anaplasma* spp./*Bartonella* spp./SFGR | 1 (0.4) | 0 (0.0) | 1 (1.1) | 1.000^c^ | 0.468^c^ |
| *Anaplasma* spp./*Coxiella* spp./*Ehrlichia* spp. | 4 (1.6) | 3 (1.2) | 2 (2.2) | 1.000^c^ | 0.663^c^ |
| *Anaplasma* spp./*Ehrlichia* spp./SFGR | 6 (2.5) | 1 (0.4) | 1 (1.1) | 0.122^c^ | 0.679^c^ |
| *Bartonella* spp./*Coxiella* spp./*Ehrlichia* spp. | 1 (0.4) | 0 (0.0) | 1 (1.1) | 1.000^c^ | 0.468^c^ |
| *Bartonella* spp./*Coxiella* spp./SFGR | 1 (0.4) | 1 (0.4) | 0 (0.0) | 1.000^c^ | 1.000^c^ |
| *Bartonella* spp./*Ehrlichia* spp./SFGR | 3 (1.2) | 0 (0.0) | 1 (1.1) | 0.249^c^ | 1.000^c^ |
| Four TBPs |  |  |  |  |  |
| *Anaplasma* spp./*Bartonella* spp./*Ehrlichia* spp./  SFGR | 1 (0.4) | 0 (0.0) | 1 (1.1) | 1.000^c^ | 0.468^c^ |

Values are n (%). ^a^: compared between tick bite group and no tick bite group. ^b^: compared between tick bite group and SFTS patients. ^c^: Analyses were performed using Fisher’s exact test. Abbreviations: SFGR: Spotted fever group rickettsiae; TBPs: Tick-boren pathogens.
